# Autoimmune Hemolytic Anemia in a Renal Transplant Patient Following Seasonal Influenza Vaccination

**DOI:** 10.1155/2019/3537418

**Published:** 2019-10-20

**Authors:** Imran Gani, George Hinnant, Rajan Kapoor, Natasha Savage

**Affiliations:** ^1^Department of Nephrology, Hypertension and Transplant Medicine, Augusta University Health, Augusta, Georgia; ^2^Department of Pathology, Augusta University Health, Augusta, Georgia

## Abstract

Vaccines aim to prevent disease occurrence, its severity, and resultant complications. Our patient, a 58-year-old male, received seasonal influenza vaccination as part of routine health maintenance. Three days later, he presented with malaise, fever, and yellowish discoloration of eyes. His labs showed hyperbilirubinemia, anemia, elevated lactate dehydrogenase, and low haptoglobin, consistent with hemolytic anemia. Autoimmune hemolytic anemia has been associated with vaccine use and may result from phenomena of molecular mimicry and cross-reactivity with the possible role of vaccine adjuvants as well. An underlying structural defect of the red blood cell membrane may make them prone to hemolysis. The differential diagnosis and work-up of hemolytic anemia is extensive, as performed in our case. Management strategies for vaccine-induced hemolysis may involve supportive care, red blood cell transfusion, steroids, and intravenous immunoglobulin.

## 1. Introduction

Seasonal influenza vaccine aims to protect against infection by influenza virus and resultant complications. Vaccines have been associated with autoimmune phenomena including triggering of autoimmune hemolytic anemias. Hemolytic anemia can present as chronic anemia secondary to chronic low-grade hemolysis or as brisk hemolysis leading to frank anemia that requires prompt medical treatment. Herein, we present a unique case of acute on chronic hemolytic anemia after a routine influenza vaccine in a kidney transplant patient.

## 2. Case Presentation

A 58-year-old Caucasian male with a past medical history of end-stage renal disease secondary to hypertension, hyperlipidemia, and diabetes mellitus type 2, received a living related kidney transplant in 1994. He was in his routine state of health with stable allograft function and was seen in internal medicine clinic for health maintenance visit where he received a seasonal influenza vaccine (0.5 ml intramuscular in the deltoid in October 2018—Quadrivalent Inactivated Influenza Vaccine IIV4). Three days later, he presented with malaise, chills, fever (up to 101.6 F), and yellowish discoloration of eyes. Physical examination confirmed icteric sclerae. His labs were significant for hemoglobin of 12.5 g/dl (baseline hemoglobin of approximately 14.4 g/dl), total bilirubin of 5.1 mg/dl, and mildly elevated AST, ALT, and ALP. Platelet count and serum creatinine were normal. LDH was elevated and haptoglobin was very low with an elevated reticulocyte percentage of 4.2%. He denied any new medications, history of allergy, or any autoimmune disease. Rapid flu test was negative for both influenza A and B. The clinical picture and labs were suggestive of hemolytic anemia post routine influenza vaccine.

On further work-up, blood culture, urine culture, and acute viral hepatitis panel for HBV, HCV, and HAV were negative. The conventional tube technique direct antiglobulin test (DAT)/Coombs test was negative. The Coombs test was performed by using a polyspecific antihuman globulin reagent and monospecific antibodies to IgG and C3d. Parvovirus B19, Epstein–Barr virus, and cytomegalovirus PCR were also negative. Iron studies and ferritin levels were normal. G6PD levels and pyruvate kinase levels were within normal range. Hemoglobin high-performance liquid chromatography (HPLC) was unremarkable. A right upper quadrant ultrasound demonstrated borderline hepatomegaly along with gallstones with no acute pathology.

**Figure 1:**
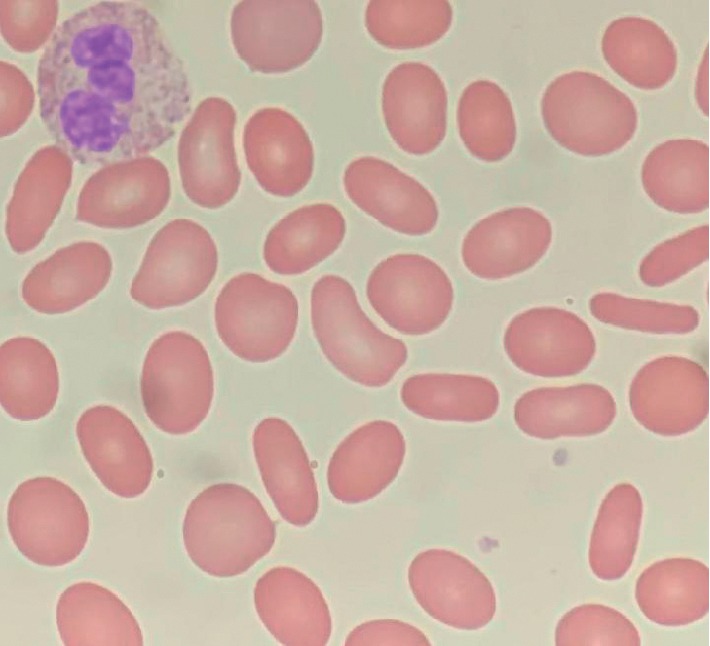
Morphologic review of the Wright–Giemsa stained peripheral blood smear revealed normochromic, normocytic red blood cells with anisopoikilocytosis. Many red blood cells (>25%) were elliptocytes. Elliptocytes are shaped like a pencil or thin cigar, with blunt ends and parallel sides.

On review of the patient's labs and clinical records, the patient had been having low degree hyperbilirubinemia since 2003 (Bilirubin 1.3–1.8). He had elliptocytes on red cell morphologic review in 2004 and 2007 that had been overlooked. He did not carry a formal diagnosis of any structural erythrocyte abnormality. A peripheral blood smear was ordered during his acute hemolytic event. It demonstrated normochromic and normocytic red blood cells. However, there was significant anisopoikilocytosis with a predominance of elliptocytes suggestive of hereditary elliptocytosis (Figure 1).

## 3. Outcome and Follow-Up

The patient was managed conservatively with supportive care. As he was in a compensated hemolytic state, he did not require any blood transfusion. No steroids or intravenous immunoglobulin were used. Folic acid supplementation was started. Avoidance of hemolysis causing drugs in the future was suggested.

On follow-up, the patient had mild persistent anemia (hemoglobin 13.1–13.5 mg/dl) and resolving hyperbilirubinemia which stabilized close to his baseline bilirubin (1.9 mg/dl). His transaminitis resolved completely.

## 4. Discussion

Influenza vaccination can lower the risk of influenza and its complications. Kidney transplant patients, because of their iatrogenic immunosuppression, are at a higher risk of such infectious complications. Our patient received a quadrivalent, egg-grown, inactivated influenza vaccine and presented with hemolytic anemia 3 days later. Hemolytic anemia can occur after preventive vaccination. Influenza vaccine has been infrequently associated with autoimmune hemolytic anemia (AIHA). The exact mechanism of AIHA induced by vaccines is unknown. Molecular mimicry of host antigens by viral peptides inducing cross-reactivity by T-cells and B-cells has been proposed. Possible role of vaccine adjuvants has also been proposed [1].

There have been case reports of AIHA occurring a few days after influenza vaccination [2]. Other autoimmune manifestations after influenza vaccination reported include thrombocytopenia, vasculitis, and Guillain–Barré syndrome. Montagnani et al. reported two cases of AIHA after influenza vaccination. In the first case, an 83-year-old woman developed Coombs-positive AIHA two days after she received an influenza vaccine. The patient was treated with steroids and immunoglobulin. The second case describes a 74-year-old woman with underlying aortic valve disease who developed Coombs-positive AIHA approximately three days after she received an influenza vaccine. The patient died two days later after being hospitalized despite treatment with corticosteroids and blood transfusion [1]. In a case report by Shlamovitz and Johar a 50-year-old man with no past medical history developed Coombs-positive Evans syndrome (i.e., AIHA with autoimmune thrombocytopenia) four days after receiving influenza vaccine that responded to treatment with steroids and immunoglobulin [3]. A nine-year-old boy developed Coombs- negative autoimmune hemolytic anemia five days after receiving influenza vaccination twice. The anemia was severe and necessitated blood transfusion, but the patient eventually recovered without additional treatment [4].

Hemolytic anemias are broadly characterized as intrinsic and extrinsic (i.e., due to intrinsic disorders within the red blood cells or extrinsic to the red cell) or divided as intravascular or extravascular. Intrinsic hemolytic anemias include membrane disorders, enzyme deficiencies, hemoglobinopathies/thalassemias, and the only acquired intrinsic form, paroxysmal nocturnal hemoglobinuria. Extrinsic hemolytic anemias can be broadly divided into immune or nonimmune-mediated destruction of red blood cells.

Classic laboratory findings suggesting hemolysis are low haptoglobin level, elevated LDH, elevated bilirubin, and reticulocytosis [5]. Our patient had all these findings on laboratory work. When hemolytic anemia is suspected, the DAT/Coombs test and review of the peripheral smear are vital to deriving the correct diagnosis. A positive DAT/Coombs test is classic for an autoimmune facilitated process resulting from either a primary disorder or secondary to drugs, infections, or malignancy. However, Coombs- negative autoimmune hemolytic anemia is a well-described entity; therefore, immune-mediated hemolysis cannot always be ruled out when the Coombs test is negative [6]. 5–10% of all autoimmune hemolytic anemias are direct antiglobulin test negative [7]. The negative Coombs test may result from a lower number of IgG molecules per RBC, non-IgG immunoglobulin on the RBC (IgA, rarely IgM), or low-affinity autoantibodies [8]. Moreover, a positive DAT/Coombs test is not always associated with AIHA [9]. In patients where there is a high index of suspicion for AIHA despite a negative Coombs test, “Super Coombs” test is available at reference laboratories. Patients with DAT/Coombs-negative AIHA may suffer milder anemia and hemolysis as compared to patients with DAT/Coombs-positive AIHA [10].

A negative DAT/Coombs in the presence of hemolysis may suggest an intrinsic erythrocyte defect of hemoglobin synthesis, enzymes, or membrane disorders. It may also suggest a nonimmune-mediated extrinsic hemolysis, which would include microangiopathic hemolytic anemia among other entities. The distinctive morphologic appearance of the red blood cells on a peripheral smear may be supportive of a specific diagnosis.

Initial work-up of anemia may include an iron profile as iron deficiency is a common cause of anemia, particularly when microcytic. To complete the work-up of anemia, hyperbilirubinemia, and hemolysis, exclusion of hepatitis with acute viral hepatitis panel may be appropriate as hemolysis can be seen in this setting. In our patient, hepatitis was excluded. Polymerase chain reaction for Parvovirus, Epstein–Barr virus, and cytomegalovirus was negative ruling out the other more common viral causes. Next, intrinsic causes of hemolysis such as hemoglobinopathies/thalassemias can be investigated via HPLC or hemoglobin electrophoresis. In our patient, hemoglobinopathy/thalassemia was ruled out by a normal HPLC. Also, the absence of microcytosis made thalassemia diagnosis unlikely. This is particularly important as cases of alpha thalassemia with only 1 or 2 gene deletions will have a normal HPLC. A common enzyme deficiency was ruled out by normal G6PD and pyruvate kinase levels. However, it is important to note that G6PD levels in a G6PD-deficient patient may be normal during acute hemolysis, necessitating repeat testing once hemolysis has subsided. A peripheral blood smear review by a pathologist is critical to further help diagnosis and subclassify hemolysis. This test should be utilized early on as it is potentially a cost-saving technique where the pathologist can suggest a possible cause and direct further testing. In our patient, this revealed prominent elliptocytosis.

Hereditary elliptocytosis, also called as ovalocytosis, is an inherited disorder in which the red blood cells are oval or elliptical rather than biconcave. It is caused by mutations in various genes eventually affecting the integrity of red blood cell membrane proteins and cytoskeleton. This leads to diminished mechanical stability of the RBC membrane making them prone to hemolysis. Most cases of hereditary elliptocytosis are due to mutations in the *alpha-spectrin, beta-spectrin*, or *EPB41* genes. Diagnosis is usually made by review of the peripheral blood smear. The majority of patients with elliptocytosis are diagnosed incidentally as the disease is largely asymptomatic, as seen in our patient [11, 12]. Decompensated hemolysis resulting in symptomatic anemia may occur during acute illness or other conditions that impact red cell survival. This can be more severe in patients with underlying chronic hemolysis. In our patient, the influenza vaccine was the most likely culprit.

Piecing this together, the patient had an underlying hereditary elliptocytosis/ovalocytosis and was prone to chronic hemolysis due to diminished mechanical stability of the red blood cell membrane. Chronic low-degree persistent hyperbilirubinemia supported this diagnosis. In addition, the patient potentially developed an acute hemolytic insult from recent influenza vaccination.

Our patient was diagnosed with acute on chronic hemolytic anemia secondary to influenza vaccine with underlying hereditary elliptocytosis. A right upper quadrant ultrasound showed gallstones, which can be seen in patients with chronic hemolytic disorders (pigment stones), further supporting this diagnosis. In addition, the previously elevated bilirubin levels as well as prior noted elliptocytosis support this entity. The concordant acute AIHA diagnosis was supported by the development of hemolysis within a short time (3 days) after administration of influenza vaccine, absence of any new drug intake, and an extensive and negative work-up as mentioned above. The Naranjo algorithm used for determining the likelihood of adverse drug reaction due to the suspected drug categorized the event as “probable” adverse drug event. Of course, the elevated LDH, reticulocytosis, and suppressed haptoglobin support hemolysis in general.

## 5. Conclusion

Influenza vaccination may infrequently set off an episode of AIHA. The exact etiopathogenesis of such an event is not clearly understood. Molecular mimicry and role of vaccine adjuvants and other constituents have been proposed. An underlying erythrocyte membrane defect may act as a risk factor. Patients who receive an influenza vaccine should be counseled and educated about reporting any unusual signs and symptoms experienced after the vaccination.

## References

[1] Montagnani S., Tuccori M., Lombardo G. (2011). Autoimmune hemolytic anemia following MF59-adjuvanted influenza vaccine administration: a report of two cases. *Annals of Pharmacotherapy*.

[2] Shizuma T. (2014). Autoimmune hemolytic anemia following influenza virus infection or administration of influenza vaccine. *Journal of Blood Disorders & Transfusion*.

[3] Shlamovitz G. Z., Johar S. (2013). A case of Evans’ syndrome following influenza vaccine. *The Journal of Emergency Medicine*.

[4] Tsuchiya H., Ishii T., Fujiwara H., Matsuda I. (1986). A case of Coombs-negative autoimmune hemolytic anemia, possibly caused by influenza vaccination. *Pediatrics International*.

[5] Luzzatto L., Jameson J., Fauci A. S., Kasper D. L. (2019). Hemolytic anemias. *Harrison’s Principles of Internal Medicine, 20e*.

[6] Takahashi T. (2018). Direct antiglobulin test-negative autoimmune hemolytic anemia. *Acta Haematologica*.

[7] Garratty G. (2005). Immune hemolytic anemia associated with negative routine serology. *Seminars in Hematology*.

[8] Segel G. B., Lichtman M. A. (2014). Direct antiglobulin (“Coombs”) test-negative autoimmune hemolyticanemia: a review. *Blood Cells, Molecules and Diseases*.

[9] Salama A. (2015). Clinically and/or serologically misleading findings surrounding immune haemolytic anaemias. *Transfusion Medicine and Hemotherapy*.

[10] Kamesaki T., Toyotsuji T., Kajii E. (2013). Characterization of direct antiglobulin test-negative autoimmune hemolytic anemia: a study of 154 cases. *American Journal of Hematology*.

[11] Coetzer T. L., Kaushansky K., Lichtman M. A., Prchal J. T. (2019). Erythrocyte membrane disorders. *Williams Hematology, 9e*.

[12] Bunn H., Lux S. E., Aster J. C., Bunn H. (2017). Inherited hemolytic disorders of the red cell membrane and red cell metabolism. *Pathophysiology of Blood Disorders, 2e*.

